# Optimising the mainstreaming of renal genomics: Complementing empirical and theoretical strategies for implementation

**DOI:** 10.1038/s41431-025-01797-x

**Published:** 2025-02-12

**Authors:** Lin Cheng, Nathasha Kugenthiran, Catherine Quinlan, Zornitza Stark, Kushani Jayasinghe, Stephanie Best

**Affiliations:** 1 Melbourne School of Health Sciences, Faculty of Medicine, Dentistry and Health Sciences, The University of Melbourne, Parkville, VIC Australia; 2 Kidney Regeneration, Murdoch Children’s Research Institute, Melbourne, VIC Australia; 3 Genomics in Society, Murdoch Children’s Research Institute, Melbourne, VIC Australia; 4 Department of Paediatric Nephrology, Royal Children’s Hospital, Melbourne, VIC Australia; 5 Department of Paediatrics, Faculty of Medicine, Dentistry and Health Sciences, The University of Melbourne, Melbourne, VIC Australia; 6 Australian Genomics, Murdoch Children’s Research Institute, Parkville, VIC Australia; 7 Department of Nephrology, Monash Health, Clayton, VIC Australia; 8 School of Medicine, Monash University, Melbourne, VIC Australia; 9 Department of Health Services Research, Peter MacCallum Cancer Centre, Parkville, VIC Australia

**Keywords:** Health care, Genetic services

## Abstract

To identify and develop complementary implementation strategies that support nephrologists in mainstreaming renal genomic testing. Interviews were conducted with individuals nominated as ‘genomics champions’ and ‘embedded genomics experts’ as part of a mainstreaming project to identify initial barriers and investigate empirical strategies for delivering the project at initial stage. Data were mapped onto implementation science framework to identify complementary theoretical strategies. Interviews with 14 genomics champions and embedded genomics experts (genetic counsellors, nephrologists, renal nurses), identified 34 barriers to incorporating genomic testing into routine care, e.g., lack of long-term multidisciplinary team support and role clarity. In total, 25 *empirical implementation strategies* were identified such as creating new clinical teams. Using the Consolidated Framework for Implementation Research, 10 complementary *theoretical implementation strategies* were identified. Our study presents a novel approach complementing empirical strategies with theoretical strategies to support nephrologists in incorporating genomic testing into routine practice. Complementary strategies can potentially address barriers and inform future studies when mainstreaming renal genomics. This process underscored the need for integrating collaborative efforts among health professionals, patients, implementation scientists and the health system to overcome identified challenges to mainstream genomic testing. Future research should explore the applicability of these strategies to support mainstreaming genomic testing in different clinical settings.

## Introduction

Genomic testing is increasingly incorporated into healthcare, including the care for patients with genetic kidney diseases [1], where it has clearly demonstrated value in terms of diagnostic utility and cost-effectiveness [2–5].

In Australia, a successful national kidney genetics collaborative, KidGen has established a network of multi-disciplinary kidney genetic clinics to support the delivery of genomic testing [1, 6]. Although this model of care has established a high diagnostic yield and clinical utility of genomic testing [6, 7], the waiting time for testing and return of results has grown, due to the recognition of the impact of genomic testing and subsequent increase in demand.

Building on formative research, we sought to address these challenges through the implementation of a Clinical Change Program in the state of Victoria [6, 7]. We developed a new hub-and-spoke delivery model for the Clinical Change Program to support broader access to genomic testing. In this program, four tertiary hospitals serve as the ‘hub’. These hubs maintain existing referral pathways and manage patients requiring multidisciplinary review while acting as central contact points for renal genomics support. They facilitate central multidisciplinary team (MDT) meetings to assist nephrologists with patient selection and result interpretation. The ‘spoke’ consists of peripheral hospital site nephrologists, who can attend central MDT meetings and -receive support from genomics champions and embedded genomics experts, allowing local nephrologists to initiate genomic testing. Genomics champions and embedded genomics experts were recruited from hospitals in Melbourne, Australia, that already offer genomic testing. In our Clinical Change Program, the genomics champions are nephrologists and renal nurses, while the embedded genomics experts include clinical geneticists and genetic counsellors.

Implementation strategies in clinical settings are frequently developed based on clinicians’ intuition. However, this approach often lacks a theoretical foundation which can lead to problems in replicability and generalisability of an intervention [8]. To address this issue, employing implementation science theory can be beneficial. By aligning clinicians’ intuition with implementation science theory, models or frameworks, complementary implementation strategies can be generated and generalised in various settings [8]. Implementation science theory, models and frameworks have been employed in genetics, and previous studies have demonstrated their value in supporting the uptake of clinical genomic testing and ensuring health services are provided and improved to meet patient needs [1, 9–12].

To investigate the mainstreaming of kidney genomics, we employed an implementation science framework, the Consolidated Framework for Implementation Research (CFIR) [13, 14], to structure an investigation into the factors influencing the Clinical Change Program’s uptake and generate implementation strategies to support nephrologists. The CFIR is a comprehensive framework, commonly used in genomic implementation research to systematically assess factors that influence the implementation of interventions in healthcare settings [15–17]. There are five domains: the intervention characteristics; outer setting domain; inner setting domain; characteristics of individuals; and the implementation process [13, 14].

In this study, we had three aims: i. to identify barriers to delivering the Clinical Change Program aimed at mainstreaming renal genomics, ii. to identify clinicians’ empirical implementation strategies for implementing the program, and iii. to generate theory-informed implementation strategies to support mainstreaming of kidney genomics. *Empirical* implementation strategies in our study refer to those informed by clinicians’ intuition and their practical clinical experiences to overcome barriers in implementing the Clinical Change Program. In contrast, *theoretical* implementation strategies are derived from implementation science theories, models, or frameworks.

## Subjects and methods

### Participants

Genomics champions and embedded genomics experts played significant roles in upskilling nephrologists to incorporate genomic testing into routine care. Specifically, they were responsible for upskilling nephrologists by attending multidisciplinary team (MDT) meetings, providing education, and ad hoc advice as required. The genomics champions and embedded genomics experts were also upskilled in kidney genomics, with increased exposure to this subspecialty and support from the Clinical Change Program and network of clinicians. They were invited to participate in semi-structured interviews at the beginning of the Clinical Change Program to investigate perceived initial barriers and enablers to the mainstreaming of renal genomics through the implementation process.

### Study design and data analysis

We used a cross-sectional, qualitative study design with semi-structured interviews. The interview guide was informed by the CFIR [13, 14] and included questions such as, “In our project, is there anything particularly helpful for your work in using genomics?” to measure the inner setting domain of CFIR, and question “Before joining our project, how confident did you feel when talking about renal genomics with patients?” to measure “characteristics of individuals”. One experienced researcher (LC) conducted the interviews either online or in person, according to participant preference. Interviews lasted 25–45 min. All interviews were audio recorded and transcribed verbatim for data analysis. The interviews were conducted between April and October 2023.

Transcripts were analysed using both inductive and deductive approaches. First, to identify barriers and enablers perceived by clinicians, two implementation scientists (LC and SB) extracted barriers and enablers to the mainstreaming of renal genomics through the Clinical Change Program. Three transcripts were randomly selected and coded independently. Extracted barriers and enablers were discussed to reach a consensus during regular fortnightly meetings. LC then completed the extraction of the barriers and enablers from the remaining transcripts, with ongoing discussions with SB to finalise the full list of barriers and enablers.

**Fig. 1:**
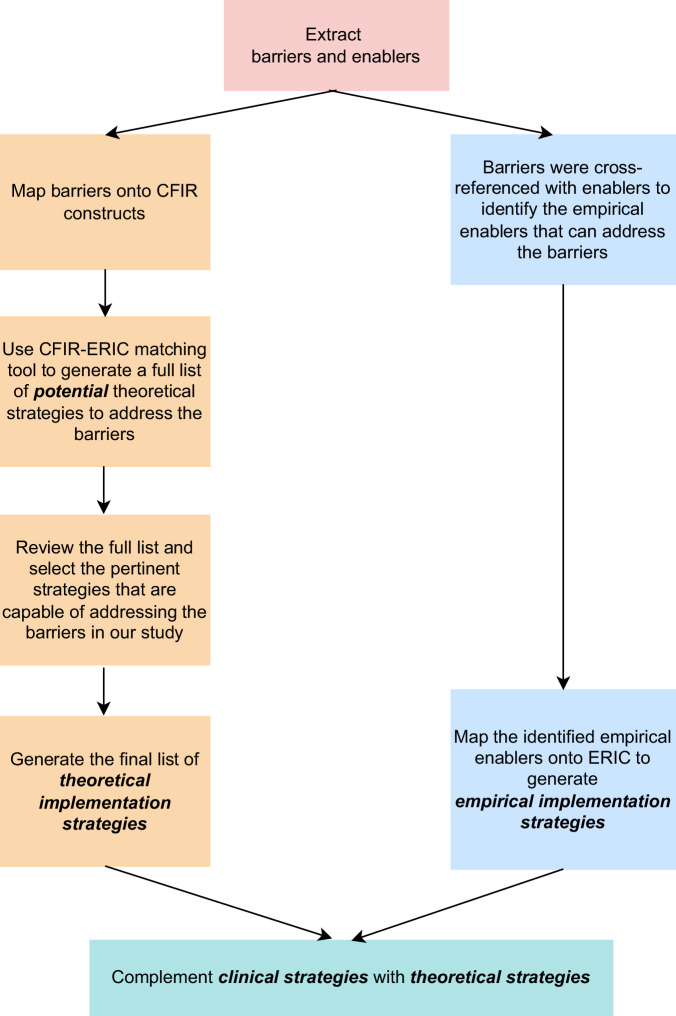
Process for developing implementation strategies. This figure illustrates how implementation strategies were developed using ERIC for mainstreaming the renal genomics.

These barriers informed the development of *theoretical implementation strategies*, and enablers informed the identification of *empirical implementation strategies*. The enablers identified by genomics champions and embedded genomics experts were defined as *‘empirical implementation strategies*’ in this study as they were based on the genomics champions’ and embedded genomics experts’ clinical experience. To complement these experiential strategies, ‘*theoretical implementation strategies’* were developed by mapping interview data onto implementation science framework. Figure 1 outlines the process of generating these strategies for mainstreaming renal genomics through the Clinical Change Program.

To develop *theoretical implementation strategies*, all identified barriers were mapped onto CFIR constructs to facilitate the next step using the CFIR-ERIC matching tool [18]. This tool comprises two components: (1) CFIR, identifying barriers; and (2) Expert Recommendations for Implementing Change (ERIC), generating implementation strategies. This analysis was conducted collaboratively by two implementation scientists (LC and NK) through four iterative discussions, reaching a consensus on CFIR constructs. After employing the CFIR-ERIC matching tool, the tool generated a list of theoretical implementation strategies to address the barriers. LC and NK then reviewed the list to select pertinent implementation strategies capable of addressing the challenges articulated by genomics champions and embedded genomics experts.

Second, to generate *empirical implementation strategies*, two implementation scientists (LC and NK) cross-referenced barriers with enablers to identify potential solutions from the enablers that could address the barriers raised by genomics champions and embedded genomics experts. These empirical enablers were based on genomics champions and embedded genomics experts’ reflections on their clinical experience. After achieving a consensus on the list of empirical enablers through two discussions, LC – in discussion with NK and SB – mapped these empirical enablers with ERIC strategies to conceptualise them and subsequently aligned the *empirical* and *theoretical implementation strategies* to generate a complementary set of implementation strategies.

### Ethics approval

This study was approved by the Human Research Ethics Committee of the Melbourne Genomics Health Alliance (Ethics approval number: HREC/80793/MH-2021).

## Results

A total of 14 genomics champions and embedded genomics experts from four hospital sites were interviewed: nephrologists (*n* = 3), clinical geneticists (*n* = 2), genetic counsellors (*n* = 6) and renal nurses (*n* = 3) (Table 1). Data analysis was conducted concurrently with the interviews, and after 10 interviews no new data was identified. A further four interviews were conducted to ensure a sufficient sample size had been reached [19].

**Table 1:** Demographic information.

		Clinical sites			
		Monash Health	Austin Health	Royal Melbourne Hospital	Royal Children’s Hospital
Professional background	Nephrologists	1	/	/	2
	Clinical geneticists	/	1	1	/
	Genetic counsellors	1	2	1	2
	Renal nurses	1	/	1	1
Number of participants = 14					

### Barriers to delivering the Clinical Change Program to mainstream genomics

A total of 102 quotes were extracted, identifying 34 barriers. The most frequently mentioned barriers were that nephrologists lacked time to incorporate genomic information into routine care and did not feel confident in discussing genomic testing with patients. The other frequently mentioned barriers were about the operation of the Clinical Change Program, such as role clarity for genomics champions and embedded genomics experts. Specifically, nephrologists and renal nurses, serving as genomics champions, were unclear about their responsibilities in delivering the Clinical Change Program. Meanwhile, geneticists and genetic counsellors were not performing their typical roles as they would in a traditional genetics setting. Instead, their roles were more varied and extended beyond their usual roles. Also, limited dissemination of the new model was highlighted as a significant barrier, potentially hindering its mainstream adoption in nephrology departments across the state. Another barrier related to the sustainability of the Clinical Change Program with participants concerned about the role of genomics champions and embedded genomics experts after completion of the Clinical Change Program.

Specific barriers relevant to the initial stages of the program were identified. For example, low engagement among nephrologists was noted at the beginning of the Clinical Change Program’s implementation. Other perceived barriers included integrating the Clinical Change Program into existing clinical workflows, a lack of working space for genetic counsellors within nephrology departments, and the additional workload for renal nurses to prepare documents for case review in MDT meetings and gather patients’ information for genomic testing. Table 2 shows the full list of perceived barriers at the beginning of the Clinical Change Program.

**Table 2:** Barriers to the delivery of the Clinical Change Program.

Barriers	Example quotes
Challenging to create a logistically simple framework for mainstreaming	“One is creating a framework where it is logistically simple enough for the clinicians to actually deliver the testing that it will actually be taken up.”
Challenging to engage nephrologists outside the hospital	“It remains to be seen how much engagement we get from nephrologists outside of our hospital. I hope we do but I guess I can anticipate that being a bit of a challenge because we don’t have a direct link there.”
Challenging to have a follow-up process with patients	“I think it is the logistics challenges sometimes. For example, patients would want time to think about whether or not they want genomic testing and their next appointment might be in about 6 months, so whether or not we should be including some kind of follow up or not or letting them go away and contact us if they want to get testing started or if they are happy to wait 6 months to get started. I think that could be challenging including like creating a process for that within this model.”
Challenging to introduce a new intervention into the current clinical workflow	“I expect there to be some resistance or hesitancy from the nephrologists. This is a new process for them.”
Difficult to manage patients with current insignificant results	“I guess the other challenge would be, if the results that come through for example are of insignificance, or they fall under the classs-3 which you don’t know how significant that result is, then the challenge will probably be like in the clinical scenario and the test result in terms of – is this a patient then you have to revisit in 2–3 years’ time when genes have been identified and you need to go through that testing again.”
Discomfort with champion title	“No I don’t think I have ever called it (genomic champion) that actually. I don’t refer to myself like that. I think of myself as a genetic counsellor embedded within the nephrology department supporting a mainstreaming project, so I guess I don’t call it that so I don’t know that my colleagues would think of that way either or calling it that.”
Disseminate the model of care	“I don’t think that the referring nephrologists really know the model yet but that’s obviously the next step to start increasing their awareness of this project and the people involved so that they know who to contact.”
Equity of accessing the care	“That relate to equity of access and equity of care in terms of what happens in a controlled environment in one large paediatric hospital Vs. what’s happening across the entire nephrology specialty – publics, private, adult, paediatrics; all those sorts of things, so I worry about that.”
Expensive to use genomic testing widely	“I thought it was financial… I think the price has come down for the testing now. I think that was the biggest barrier, I thought.”
Extra workload	“That would be done by the geneticists at the moment so that’s about transferring that workload back into the nephrology clinic which is going to impact on patient’s time.”
Feel overwhelming as a lot of stuff to learn	“It sometimes feels very overwhelming because there is a lot of stuff you need to know.”
Insufficient communication skills to consent patients	“I also think that communication skills; like being able to translate pretty complex information and be a skilled communicator is a huge skill that’s of benefit to do this sort of work… I think there are risks in the very quick discussion with a nephrologist, in that patients and families trust them and maybe are willing to yet to what the nephrologist suggests.”
Insufficient feedback from different stakeholders/settings	“I guess it is a bit tricky because I am so embedded in the whole thing… My understanding so far is that things maybe are a little bit different in the paediatric setting Vs. the adult setting for this study, so there probably will be differences in how staff are being used and how the project is running.”
Insufficient renal genetics clinic	“If we can establish a frequent nephrology genetics clinic, and that more complex patients can be going to the specialised clinic… I think that’s the hope.”
Insufficient resources for particular individual genetic conditions	“What I am thinking about is patient information sheets for particular individual genetic conditions… I am always scouring the internet for the resources similar to that already exist and trying to use them. I often find that there is very little that meets all my needs and I often kind of hack in together my own based on information I get from various places, so that’s something that I like to see available as part of this project.”
Insufficient support for using genetics	“…It would be good to get genetic and everyone is like but how do we do it.”
It takes time to upskill	“I think the biggest thing will be time for people to wrap their head around it and get comfortable to use it.”
Lack of confidence	“I would say that I am still learning a lot about renal genomics and when results coming back and they will have to start talking a lot more about renal genomics, I am a bit uncertain.”
Lack of education and upskilling for clinicians	“Mainstreaming can create its own challenges because it is outside of that well-support MDT review team often.”
Lack of long-term MDT support	“Mainstreaming can create its own challenges because it is outside of that well-support MDT review team often.”
Lack of physical space	“In a busy hospital where space is at a premium, one thing that we are already encountering is that if the genetic counsellors are roving through a nephrologist clinic – trying to offer assistance but they don’t have a room/space to work in, that is a huge challenge…”
Lack of time to deliver care	“The clinicians are having to add an extra to order and an extra consent process. Some clinicians have felt it is difficult in the time they have been allocated for an appointment.”
Learning resources are difficult	“I just do the learning package but I found it quite overwhelming to be honest, because it has been a long time since I did anything with genetics. I found that the learning packages were quite difficult”
Legitimisation within the hospital	“More wider acceptance and validation from both within the nephrology department and across the hospital would be good… I think a shift in thinking towards this is a recognised and valid part of hospital care, embedding that within the structure of the hospital and the department and for it to be visible as well and not hidden role
Limited funding	“Given the number patients that would benefit from genomic testing and the limited funding to provide that, I don’t think it is sustainable.”
Limited working time to support nephrologists’ needs	“The only concern is that if the project was successful and lots of nephrologists decide they want to do genomic testing, whether I would have the capacity within my 1 3/4 h a week to accommodate or support that.”
Long waiting time to receive genomic service	“I understand that there time barriers in terms of how long it takes to get tests back and hopefully we can reduce any extensions to that time that time has been caused by us not knowing how to do things properly.”
Low engagement	“They are happy to have the project happen but I don’t get the feeling that they are particularly driving engagement or enthusiasm for the project and otherwise not actively engaged.”
Ensure patients receive consistent information across different appointments	“I think spreading it over a few times would work but then that raises the issue of consistency. You are giving consistent information to the patient each time, so obviously they may not see the same doctor every time, so I guess that’s a challenge as well, the consistency and trying to make sure everyone is consistent with the information they give to the patient.”
New model of care not yet operationalised	“It is difficult to know as I guess for me, I am still lacking in information on exactly how it is going to be rolled out.”
Lack of feedback opportunity	“We haven’t really had an opportunity to get feedback from the about the new model…”
Organisational challenges	“The actual limitation of the hospital system to arrange for her (the genetic counsellor) to see patients outside of renal genetics clinic, that’s actually really difficult.”
Role clarity	“knowing where everyone’s role ends and knowing who is best to help in which scenario. There is a lot of unknown.”
Sceptical about the model	“I think there is quite a lot of challenges and I do already wonder if we have got the model quite right. So I think there is lots to think about, lots to try out and lots to learn from…”
To ensure the quality of patient care in mainstreaming clinics - consider the whole family	“I do also worry that because genetics is so used to seeing the family as well as the patient, I do worry that mainstreaming individualises things and removes that family aspect. Because one nephrologist is looking after one person and cares about their clinical care.”

### Empirical implementation strategies to mainstream genomics in nephrology

A total of 133 quotes were extracted from the interviews, representing 26 empirical enablers that could facilitate the delivery of the new model of care. The quotes representing empirical enablers were cross-referenced with barriers to identify potential solutions (Fig. 1). These empirical enablers were coded to the ERIC to conceptualise them as *empirical implementation strategies*. The mapping resulted in 25 *empirical implementation strategies*. The most frequently mentioned empirical strategies included ‘promote network weaving’, ‘create a learning collaborative’, ‘change physical structure and equipment’ and ‘provide clinical supervision’. Supplementary Table 1 lists the full set of *empirical implementation strategies* addressing the identified barriers.

### Complimentary theoretical implementation strategies to support mainstream genomics in nephrology

After mapping all barriers onto CFIR constructs to conceptualise the problems, the CFIR-ERIC matching tool was employed to generate *theoretical implementation strategies* to complement the *empirical implementation strategies*. This mapping resulted in 35 theoretical implementation strategies, such as ‘identify and prepare champions’, ‘facilitation’, ‘create a learning collaborative’ and ‘capture and share local knowledge’.

**Fig. 2:**
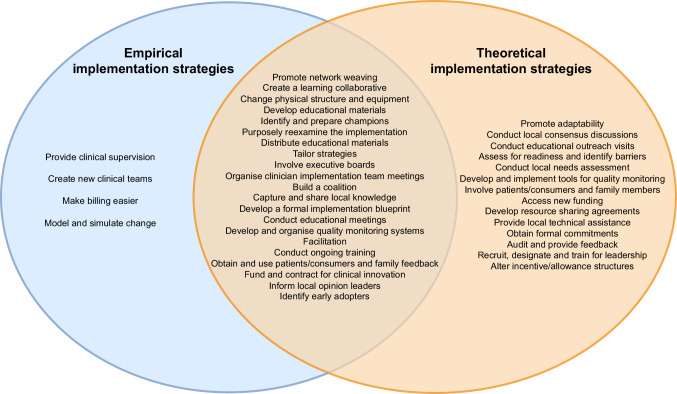
Empirical implementation strategies and theoretical implementation strategies.

When we aligned the two sets of strategies, there were some overlaps between the *empirical* and *theoretical implementation strategies*. As shown in Fig. 2, empirical implementation strategies could be supplemented by the theoretical implementation strategies. A total of 21 overlapped strategies, such as ‘promote network weaving’ and ‘create a learning collaborative’, were generated from both clinicians’ experience and implementation theory. Four empirical implementation strategies, such as ‘provide clinical supervision’ and ‘create new clinical teams’, were not identified in theory, while 14 theoretical implementation strategies, such as ‘promote adaptability’, ‘conduct local consensus discussions’ and ‘conduct educational outreach visits’, were not identified based on clinical experiences.

**Fig. 3:**
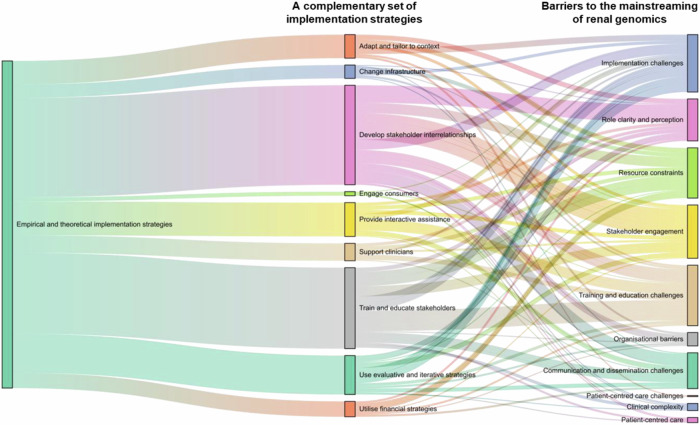
How empirical and theoretical implementation strategies can address the barriers to support the mainstreaming of renal genomics. This figure illustrates how empirical and theoretical implementation strategies shown in the middle bars can address the barriers shown in the right bars.

Figure 3 shows nine clusters of implementation strategies [20] and ten groups of barriers. It illustrates how the identified ‘barriers’ can be addressed by empirical and theoretical implementation strategies. Specific implementation strategies and barriers can be found in Supplementary Tables 2 and 3. For example, in relation to the complexity of genomic testing, the most common challenge nephrologists reported was time constraints during patient consultations. Incorporating genomics requires additional time to discuss the testing with patients and interpret test results. It can be difficult to cover large amounts of information about both nephrology and genomics in the consultation. To address this, clinicians suggested implementation strategies to ‘identify and prepare champions’ such as genetic counsellors to assist with patient communication and result interpretation outside of renal consultations. Complementary theoretical implementation strategies suggest nephrologists may consider such as ‘capture and share local knowledge’ from implementing sites to learn how other clinicians manage this challenge. Additionally, the theoretical implementation strategy, ‘promote adaptability’, indicated that the Clinical Change Program needs to be further enhanced to adapt to the identified barriers.

For the challenges related to the initial operationalising of the program to mainstream genomics, some issues such as unclear responsibilities of genomics champions and embedded genomics experts for supporting the mainstreaming of the renal genomics and low awareness of the Clinical Change Program among clinicians were identified. To clarify the genomics champions and embedded genomics experts roles, while there were several empirical implementation strategies such as ‘purposely reexamine the implementation’, theoretical implementation strategies provided additional lenses to address the problem such as ‘develop and implement tools for quality monitoring’ for the process of mainstreaming. To increase awareness of the Clinical Change Program, empirical implementation strategies like ‘promote network weaving’ and ‘create a learning collaborative’ were proposed. In addition, the theoretical implementation strategy suggested ‘organise clinician implementation team meetings’ to support clinicians mainstreaming renal genomics to reflect on the implementation effort and share learning experiences.

## Discussion

To improve equitable and timely access to genomic testing, we have implemented a Clinical Change Program to mainstream genomic testing in nephrology departments in Australia. However, an evidence-based healthcare intervention alone is insufficient to guarantee its successful implementation, as various factors in real-world settings will influence uptake [21]. Implementation science provides rigorous approaches to explore these factors and generate strategies to address the problems. By employing an implementation science framework, CFIR, we not only identified barriers reported in previous studies that hinder the mainstreaming of genomic testing in clinical practice [22, 23], but also uncovered additional barriers in real-world implementation that were previously overlooked. Subsequently, we used the CFIR-ERIC matching tool to map out the clinicians’ strategies to address the barriers to mainstreaming renal genomics. Various empirical implementation strategies were proposed by clinicians and implementation science frameworks generated complementary strategies to support nephrologists in incorporating genomics in renal healthcare.

The numerous ‘barriers’ identified demonstrate how challenging it is to incorporate genomics into nephrologists’ routine clinical practice. Our findings also indicated that the challenges stem from both the complexity of using genomics in renal healthcare and from trying to integrate a new model of care into routine care.

The barriers related to the complexity of genomic testing, such as inadequate training for nephrologists to incorporate genomic testing in standard care, translating genomic testing into personalised clinical management and the lack of necessary infrastructure for routine implementation, have been reported in previous studies [22, 23]. Clinicians have strategies to solve these problems, but they may also benefit from incorporating *theoretical implementation strategies* [8]. Our results confirmed that *empirical implementation strategies* can be complemented by *theoretical implementation strategies*. For example, one barrier was that nephrologists felt lack of confidence in incorporating genomics into their standard practice, which was also identified in a previous study [6]. While clinicians came up with the empirical implementation strategy such as ‘provide clinical supervision’ and ‘develop educational materials’, there were other complimentary theoretical implementation strategies such as ‘create a learning collaborative’ to foster a collaborative learning environment, ‘identify and prepare champions’ to help with upskilling clinicians, and ‘facilitation’ for interactive problem solving and support.

The mainstreaming of genomic testing is hindered not only by barriers related to its complexity, but also how frontline clinicians integrate genomic testing into their standard workflow [24]. This compounded challenge highlights the need for effective and rigorously tested new models of care to incorporate genomic testing into clinical practice. As we implement the Clinical Change Program to achieve this goal, it is crucial to understand the barriers related to the implementation and to identify strategies to address them. This understanding would accelerate the mainstreaming of genomic testing in nephrology departments.

Identified barriers, such as how to incorporate genomic testing into the current clinical workflows and how to create a logistically simple program for mainstreaming, provide additional perspectives to understand the challenges of incorporating genomic testing in real-world contexts. The incorporation of genomic testing requires a holistic approach that involves a dynamic interaction among health professionals, patients and the health system [25]. Therefore, it is important to develop implementation strategies with efforts from different stakeholders and understand the barriers to its implementation. To address these barriers, we employed implementation science approaches to generate *theoretical implementation strategies* in addition to *empirical implementation strategies*, as theory-driven strategies can offer additional benefits to promote clinical intervention use and improvement [8].

However, this study has limitations. Given that our findings were based on baseline interviews, the Clinical Change Program was not fully operationalised, and there were limited opportunities to provide feedback on its functioning. Consequently, additional barriers and implementation strategies may not have been identified at the baseline. Further investigation is needed to assess and enhance our Clinical Change Program, and follow-up data should be collected to identify additional potential barriers and implementation strategies as the program is rolled out. Also, the barriers reported in this paper were perceived by genomics champions and embedded genomics experts. These may differ from those experienced by nephrologists when incorporating genomic testing in routine practice. Therefore, future work is needed to explore nephrologists’ perspectives. Additionally, since the study was carried out in the nephrology setting, future studies should investigate the feasibility and effectiveness of our approach in other clinical and healthcare settings as result may not be generalisable. Furthermore, addressing the various challenges in mainstreaming genomic testing in clinical practice requires collaborative efforts among patients, health professionals, researchers and health system to mainstream genomic testing successfully.

In conclusion, our study provided a novel approach to demonstrate how implementation science frameworks can support clinicians in identifying barriers to mainstreaming renal genomics, as well as developing both empirical and theoretical implementation strategies to support mainstreaming. These barriers and implementation strategies identified may serve as valuable references for future studies as the model of care evolve and be applied in other settings.

## Supplementary information

Supplementary tables

## Data Availability

The datasets generated and/or analysed during the current study are available from the corresponding author on reasonable request.
