# Supplementary material for: Optimising the mainstreaming of renal genomics: Complementing empirical and theoretical strategies for implementation
Source: Eur J Hum Genet. 2025 Feb 12;33(3):351–9. doi: 10.1038/s41431-025-01797-x (PMC11894066; doi:10.1038/s41431-025-01797-x)
Supplement: Supplementary file 1 — Supplementary tables [file 41431_2025_1797_MOESM1_ESM.docx]

**Supplementary Table 1 Empirical strategies**

| **Strategies from ERIC** | **Example quotes** |
| --- | --- |
| Promote network weaving | “I guess what we are trying to aim to do now is increase our visibility with the nephrologists. So we have joined a few Nephrology Department meetings... We made a specific effort to attend as well and be present and try and increase our visibility for the nephrologists but I guess moving forward, we want to do that further.” |
| Create a learning collaborative | “I think we are a very good team in terms of where we all share information and we have a number of meetings; trying to make it all open and available to everybody.” |
| Change physical structure and equipment | “In Monash Health with the printed little cards with genomic champions names on them. Because I work as a nurse in the project, we made sure that everybody has a card with the people’s names on it and the phone number where they could call.” |
| Provide clinical supervision | “I have spoken to [name] last week, one of our researchers and nephrologists here and she has offered to feed more information and interesting things and things that might apply to some of the patients that we are discussing and a bit of education over that time.” |
| Develop educational materials | “There are lots of sources. The genomics website which has been really great.” |
| Create new clinical teams | “We have set up a mainstream clinic to upskill some of the nephrology fellows to train them so that they can see how we consent patients and how we talk about genetics.” |
| Identify and prepare champions | “Other genomics champions, [name], the doctor who is running the main program and also make use of some of the genetic counsellors… other nurses who are doing the same role and the doctors around as well.” |
| Identify early adopters | “I have colleagues in other areas that are working on mainstreaming. Some working on mainstreaming in cardiac, some working on mainstreaming for neurogenetic conditions. We are working through similar issues in different areas. There are some of my colleagues support mainstreaming through the oncology clinic and so we know it can be done because we have seen it done in oncology so I guess we are aware that it can be done.” |
| Purposely reexamine the implementation | “[name] is great as is our head of department. He will try and make sure that the nephrologists will follow the procedure as possible and encourage them.” |
| Distribute educational materials | “I have received some scientific studies from Journals written by nephrologists.” |
| Tailor strategies | “I think it is great that the role is a bit flexible in terms of what we can do and there is space to go – “I have got this idea lets try this” or for each site to adapt what works for them because it is not going to be exactly the same for each site how this goes.” |
| Involve executive boards | “[name], Director of Nephrology at [name of hospital] has been really supportive of me and he is the one who has promoted it and spoke to me about it as well.” |
| Organise clinician implementation team meetings | “The current barrier is the governance perspective, just obviously those things that you need to line up and have ready to go when you are doing it as part of an implementation project which is really important. We want to be capturing this and understanding this and doing it in a really informed way.” |
| Build a coalition | “I think it will be great to build up links with nephrologists outside the [name of hospital] working in other settings.” |
| Capture and share local knowledge | “I have colleagues in other areas that are working on mainstreaming. Some working on mainstreaming in cardiac, some working on mainstreaming for neurogenetic conditions. We are working through similar issues in different areas.  I think we are a very good team in terms of where we all share information and we have a number of meetings; trying to make it all open and available to everybody.” |
| Develop a formal implementation blueprint | “Things have to be streamlined and efficient. If we set up a good framework, hopefully it will be something that will become part of normal practice like ordering an x-ray or something.” |
| Conduct educational meetings | “We have a few education meetings being set up giving us the opportunities to present to the nephrologists and some of the junior registrars have also indicated interest in upskilling in renal genomics which is great.” |
| Develop and organise quality monitoring systems | “As a project, it is all a bit new and I think it is good having these check-ins along the way. If we find that something is not quite working for us, we can through it and try and find a better way and being across different site too.” |
| Facilitation | “I have spoken to [name] last week, one of our researchers and nephrologists here and she has offered to feed more information and interesting things and things that might apply to some of the patients that we are discussing and a bit of education over that time.” |
| Conduct ongoing training | “There is a weekly department meeting that’s very clinical and then they have department education meetings, which we are included in. The education is monthly but the department meeting is weekly.” |
| Obtain and use patients/consumers and family feedback | “I think the way that the resources have been put together, to try and make sure that the right test is chosen for each patient and that they have all the information that they need, I think it will be really valuable particularly for us with potential kidney donors.” |
| Make billing easier | “The testing is getting a lot easier now and probably less expensive. There is also lots of MBS Item numbers apparently that are coming out so that could make things a little bit easier to get testing done.” |
| Fund and contract for clinical innovation | “I think it (genomic testing) is something that people are aware that now the number of the tests are on Medicare but how to go about ordering it, and how to consent a patient – all those sorts of things are a big unknown and hopefully will become clearer.” |
| Model and simulate change | “If we set up a good framework, hopefully it will be something that will become part of normal practice like ordering an x-ray or something.” |
| Inform local opinion leaders | “[name] is great as is our head of department. He will try and make sure that the nephrologists will follow the procedure as possible and encourage them.” |

**Supplementary Table 2 List of complementary set of implementation strategies**

| Clusters of implementation strategies | Specific implementation strategies |
| --- | --- |
| Adapt and tailor to context | Promote adaptability  Tailor strategies |
| Change infrastructure | Change physical structure and equipment |
| Develop stakeholder interrelationships | Build a coalition  Promote network weaving  Identify and prepare champions  Capture and share local knowledge  Organise clinician implementation team meetings  Conduct local consensus discussions  Inform local opinion leaders  Identify early adopters  Involve executive boards  Obtain formal commitments  Model and simulate change  Recruit, designate and train for leadership |
| Engage consumers | Involve patients/consumers and family members |
| Provide interactive assistance | Provide clinical supervision  Facilitation  Provide local technical assistance |
| Support clinicians | Develop resource sharing agreements  Create new clinical teams |
| Train and educate stakeholders | Create a learning collaborative  Develop educational materials  Conduct educational outreach visits  Conduct educational meetings  Conduct ongoing training  Distribute educational materials  Make training dynamic |
| Use evaluative and iterative strategies | Obtain and use patients/consumers and family feedback  Conduct local needs assessment  Assess for readiness and identify barriers and facilitators  Develop and implement tools for quality monitoring  Purposely reexamine the implementation  Develop a formal implementation blueprint  Develop and organise quality monitoring systems  Audit and provide feedback |
| Utilise financial strategies | Access new funding  Fund and contract for clinical innovation  Alter incentive/allowance structures  Make billing easier |

**Supplementary Table 3 List of barriers to the mainstreaming of renal genomics**

| Groups of barriers | Specific barriers |
| --- | --- |
| Clinical complexity | Difficult to manage patients with current insignificant results  Challenging to have a follow-up process with patients |
| Communication and dissemination challenges | Disseminate the model of care  Ensure patients receive consistent information across different appointments |
| Implementation challenges | Challenging to create a logistically simple framework for mainstreaming  Challenging to introduce a new intervention into the current clinical workflow  Equity of accessing the care  Long waiting time to receive genomic service  New model of care not yet operationalised |
| Organisational barriers | Legitimisation within the hospital  Organisational challenges |
| Patient-centred care | To ensure the quality of patient care in mainstreaming clinics - consider the whole family |
| Resource constraints | Extra workload  Expensive to use genomic testing widely  Lack of long-term MDT support  Lack of time to deliver care  Lack of physical space  Limited funding  Limited working time to support nephrologists' needs  Long waiting time to receive genomic service  Insufficient renal genetics clinic  Insufficient resources for particular individual genetic conditions |
| Role clarity and perception | Discomfort with the champion title  Role clarity  Skeptical about the model |
| Stakeholder engagement | Challenging to engage nephrologists outside the hospital  Low engagement  Lack of feedback opportunity  Insufficient feedback from different stakeholders/settings |
| Support clinicians | Support clinicians |
| Training and education challenges | Feel overwhelming as a lot of stuff to learn  Insufficient communication skills to consent patients  Insufficient resources for particular individual genetic conditions  Insufficient support for using genetics  It takes time to upskill  Lack of confidence  Learning resources are difficult |
